# Cannabinoids From *Trema micranthum* (L.) Blume (Cannabaceae): A Cannabinoid Profiling System

**DOI:** 10.1002/cbdv.202500208

**Published:** 2025-05-22

**Authors:** Yasmin Cunha‐Silva, Rayssa Ribeiro, Ricardo Finotti, Gabriel Reis Alves Carneiro, Gustavo Ramalho Cardoso dos Santos, Henrique Marcelo Gualberto Pereira, Monica Costa Padilha, Valdir Florêncio Veiga‐Junior

**Affiliations:** ^1^ Chemical Engineering Section Military Institute of Engineering Rio de Janeiro Brazil; ^2^ Department of Biology Estácio de Sá University Rio de Janeiro Brazil; ^3^ Federal University of Rio de Janeiro, Chemistry Institute, Brazilian Doping Control Laboratory Rio de Janeiro Brazil

**Keywords:** cannabaceae, cannabinoid profiling system, cannabinoids, *Trema micranthum*, UHPLC‐HRMS/MS

## Abstract

*Trema micranthum* (L.) Blume is a species from the Cannabaceae botanical family, present all over the tropics. A recent study conducted by our group identified the presence of cannabidiol (CBD), Δ^9^‐tetrahydrocannabinol, cannabidiolic acid, and tetrahydrocannabinolic acid‐A in *T. micranthum* extracts. Their presence in the plant's fruit, inflorescence, and leaf extracts was detected and quantified. *T. micranthum* has been identified as a potential new source of cannabinoids such as CBD. The present work focuses on a new Cannabinoid Profiling System (CPS) to present a wide view of cannabinoid chemical composition with several compassionate and modern mass spectrometry (MS) tools. Extracts from fruits, leaves, and inflorescences were obtained using a methanol/hexane (9:1, v/v) solvent mixture. Ultra high‐performance liquid chromatography coupled with high‐resolution tandem MS using targeted and untargeted approaches were used in these approaches. CPS allowed the identification of the 26 cannabinoids in *T. micranthum* described for the first time in this plant.

## Graphical abstract

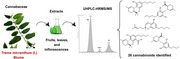

## 1 Introduction

Cannabis is the Latin term referring to the species of plants from the genus *Cannabis* (Cannabaceae). It is a highly variable, complex polymorphic species native to Central and Southern Asia. Its psychoactive potential has been known for over five millennia by the Indigenous people of the Middle East and Central Asia, historically used for recreational, medicinal, and religious purposes [1]. These effects are related to a group of substances named cannabinoids, typically present in the species *Cannabis sativa*. The two main cannabinoids that determine the pharmacological properties of Cannabis extracts are Δ^9^‐tetrahydrocannabinol (THC) and cannabidiol (CBD) [2]. THC is a psychoactive compound with euphoric, antiemetic, and analgesic properties. The controversies related to THC are considerable in society, reaching even the sports context. The use of cannabis is prohibited by the World Antidoping Agency in competition. On the other hand, CBD is a depressant and antipsychotic compound with anticonvulsant, anxiolytic, anti‐inflammatory, and antioxidant properties. Both THC and CBD are neutral substances, produced through exposure to light and heat from the decarboxylation of the originally biosynthesized carboxylic acids precursors: tetrahydrocannabinolic acid (THCA) and cannabidiolic acid (CBDA), respectively [3]. THC and CBD serve as key indicators of the potential pharmacological and toxicological properties of extracts, while their acidic precursors provide valuable parameters for assessing the integrity of the plant's raw material. As a result, monitoring these compounds is essential for ensuring quality control in the production process of cannabinoid extracts [1].

*Trema micranthum* (L.) Blume is a species also from the Cannabaceae botanical family and as the genera *Aphananthe* (Thunb.) Planch and *Celtis* L., are widely distributed in tropical and temperate regions [4, 5]. Taxonomically, the genus *Trema* has some proximity to *Cannabis*. Its congeneric species *Trema orientale* (L.) Blume is widely distributed throughout tropical Asia and is used in traditional medicine, particularly for the treatment of infectious diseases, also has cannabinoids in its inflorescence extracts [6, 7]. Cannabinoids secreting trichomes were also found for *T. micranthum* [8]. A recent study conducted by our research group investigated these two important cannabinoids and their precursors in *T. micranthum* extracts, recognizing their significant economic, medicinal, and social value [9]. Their presence in the plant's fruit, inflorescence, and leaf extracts was detected and quantified. *T. micranthum* has been identified as a potential new source of cannabinoids such as CBD [9]. The mass spectral data from that study provide compelling evidence suggesting the presence of several other cannabinoids, highlighting the need for further investigation of the extracts. Many other cannabinoids are biosynthesized in *Cannabis* species, with diverse psychoactive effects, including cannabichromene (CBC), cannabinol (CBN), cannabidivarin (CBVA), tetrahydrocannabivarin (THCV), and cannabigerol (CBG). Among these, CBN warrants special attention due to its interaction with other cannabinoids, contributing to a notable sedative effect. Additionally, it plays a pivotal role in the *entourage effect—*a synergistic relationship among the various compounds in Cannabis that amplifies the therapeutic benefits of cannabinoids [10]. Additionally, CBN is a degradation product of THC, formed through sequential decarboxylation in the presence of light, heat, and oxygen. It is interesting in quality control as a target for the degradation process and a marker of plant aging [3]. The biosynthesis of other bioactive cannabinoids with quantitative patterns distinct from those observed in *Cannabis* species could be highly intriguing from both biological and pharmacological perspectives.

In the present study, the focus was to expand the search for other cannabinoids in *T. micranthum* extracts. Interestingly, an extensive literature review revealed a lack of systematic approaches to analyzing cannabinoids using modern chromatographic and spectrometric techniques. Meanwhile, the growing commercialization and development of Cannabis‐based medicinal, cosmetic, recreational, and food products highlight the need for advanced tools. This presents a valuable opportunity to establish comprehensive, accurate, and straightforward methods for cannabinoid analysis.

There are three main approaches generally used in cannabis analysis: targeted analysis, which typically involves the use of reference materials for the identification and quantification of all analytes; non‐targeted analysis, where data mining software is used to provide an overview of the compounds present in a sample; and studies where targeted and non‐targeted approaches are used simultaneously. The difference between our work and previously published studies is the use of Product Ion Scan as a mass spectrometry (MS) experiment to infer relationships between precursor and product ions with high resolution.

In the targeted approach, high‐performance liquid chromatography (HPLC), or ultra‐HPLC (UHPLC), is typically used, coupled with quadrupole mass analyzers, either single or triple. Reference materials are used to determine the retention times of analytes, and acquisition is performed through multiple reaction monitoring, where both the precursor ion and the product ion are monitored simultaneously. This approach offers high sensitivity and selectivity; however, it does not assess any other substances present in the sample that were not included in the predefined transition list [11, 12, 13]. In the non‐targeted approach, HPLC, or UHPLC, is typically coupled with mass analyzers, such as Time‐of‐Flight (TOF) or Orbitrap, both of which operate at high mass resolution. The primary advantage of this approach is that all ionizable compounds—typically detected using electrospray ionization (ESI)—can be identified. Once acquired, the data can be reprocessed at any time in the future. Compared to triple quadrupole analyzers, this approach is less accurate when used for quantitative analysis [14, 15, 16].

The prerogative of CPS lies in the correlations that can be made through acquisition in Product Ion Scan mode, combined with the Orbitrap's vast capability for high‐resolution, non‐targeted substance acquisition, and the accuracy achievable through acquisition in parallel reaction monitoring (PRM) mode in a quantitative approach.

This article reports the cannabinoid chemo‐diversity not previously described in *T. micranthum* using UHPLC coupled with high‐resolution tandem MS (HRMS/MS) using targeted and untargeted approaches, which we called Cannabinoid Profiling System (CPS).

## 2 Results and Discussion

### 2.1 The CPS

**FIGURE 1:**
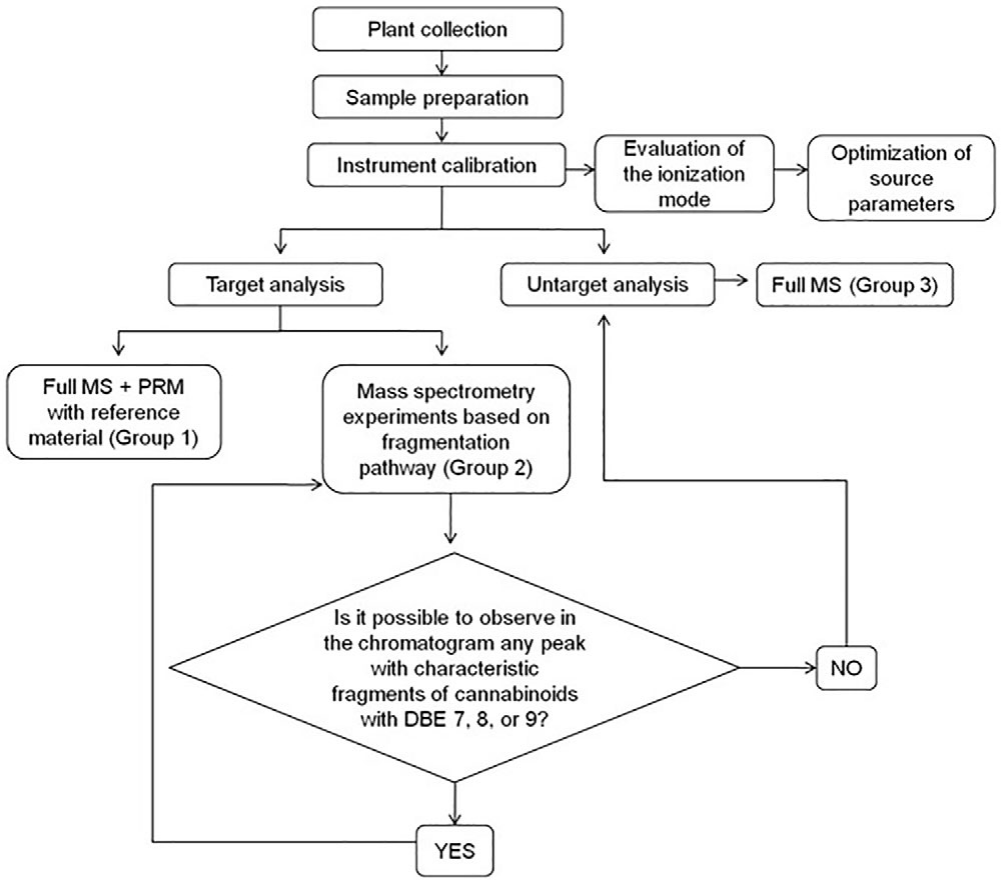
Scheme of the Cannabinoid Profiling System.

The CPS developed in this study incorporated cutting‐edge and highly precise tools to provide a comprehensive overview of metabolites across various plant parts. This approach leverages state‐of‐the‐art analytical platforms, including UHPLC‐HRMS/MS. LC‐Orbitrap instruments deliver exceptional sensitivity, selectivity, and analytical throughput, producing data structures rich in valuable information. The CPS necessarily comprises all the steps indicated in Figure 1. The first step is to evaluate the ionization of the target analytes using the proposed method. Next, two MS experiments involving PRM are performed. In the first experiment, the evaluation is compared with the reference material. The second experiment is performed through product ion scanning. The third MS experiment is conducted with Full MS acquisition, searching for cannabinoid masses described in the literature and found in *C. sativa*, which also belongs to the Cannabaceae family. For the CPS to be applied, equipment capable of operating at high mass resolution, such as Orbitrap and TOF, is required.

Previously, in the analysis of THC and CBD (and their precursors) in *T. micranthum* using HRMS/MS [9], the approach was targeting analysis, using PRM acquisition for quantification through reference material.

In the present study, the workflow includes sample preparation, and targeted and untargeted analysis, both by the high‐accuracy mass spectrometer (Figure 1). The first mass spectrometer step is to guarantee the ionization of molecules by electrospray device. After completing the initial steps, MS experiments were conducted in three parts.

The first (Group 1) consists of a targeted approach, in which the detection of specific cannabinoids becomes possible through comparison with the reference material data (Figure 1). The strategy is based on comparing the retention times and relative abundance of diagnostic ions obtained by PRM acquisition to determine the presence or absence of the target analyte in the extracts from different parts of the plant. The second part (Group 2) involves the search for cannabinoids based on the characteristic fragments for specific types of cannabinoids, using PRM as a product ion scan‐like experiment in the quadrupole‐orbitrap mass spectrometer. Finally, in the last part (Group 3), literature data were used to search for cannabinoids using Full MS, based on exact mass.

PRM was used as the acquisition mode for the first two parts of the CPS. Targeted MS‐based approaches, more specifically the PRM performed on quadrupole‐orbitrap mass spectrometers, leveraging the high resolution and trapping capabilities of the instrument, offer a clear advantage over the conventional selected reaction monitoring measurements executed on triple quadrupole instruments. PRM experiments are typically performed on the latest generation hybrid instruments, namely the quadrupole TOF (Q‐TOF) and the quadrupole‐orbitrap mass spectrometers. Such instruments exhibit high analytical performance, especially in complex samples requiring selectivity to distinguish analyte fragment ions from background signals. During PRM analyses, full MS/MS spectra were acquired, and only limited information regarding the targeted analytes (precursor ion m/z and optionally expected elution time range) was necessary before acquisition. In addition, the acquisition method required the definition of specific instrumental parameters, including the quadrupole isolation window width, the maximum fill time, and the Orbitrap resolving power. Similar to all targeted experiments, in PRM analysis, the predefined precursor ions were isolated during their monitored chromatographic elution, in the quadrupole mass filter based on the preset isolation window width. Then, the precursor ions were transferred into the collision cell and fragmented at the optimized for each precursor collision energy. The duration of this process was controlled by the automatic gain control and the maximum fill time. The resulting fragment ions were transferred back to the C‐trap and pushed to the Orbitrap for mass analysis as previously described [17]. Furthermore, the MS/MS fragmentation patterns were employed to confirm the identification of the analytes in the initial stage, while quantification was performed on the traces extracted from those fragments yielding the highest sensitivity and selectivity. Collision‐induced Dissociation experiments were performed to identify specific fragments for the cannabinoid reference material and to corroborate the data published previously in the literature [18, 19, 20].

### 2.2 Cannabinoids detected in *T. micranthum*

The first description of cannabinoids in *Trema* was performed in a study with *T. orientalis*, where TCH, CBD, and CBN were identified using reverse‐phase HPLC and gas chromatography coupled with MS (GCMS) [6]. Previously, THC and CBD were identified in *T. micranthum*, together with their acid precursors [9]. In the present study, the extracts (methanol/hexane 9:1) from *T. micranthum* fruits, inflorescences, and leaves were analyzed by UHPLC‐HRMS/MS using the CPS.

**FIGURE 2:**
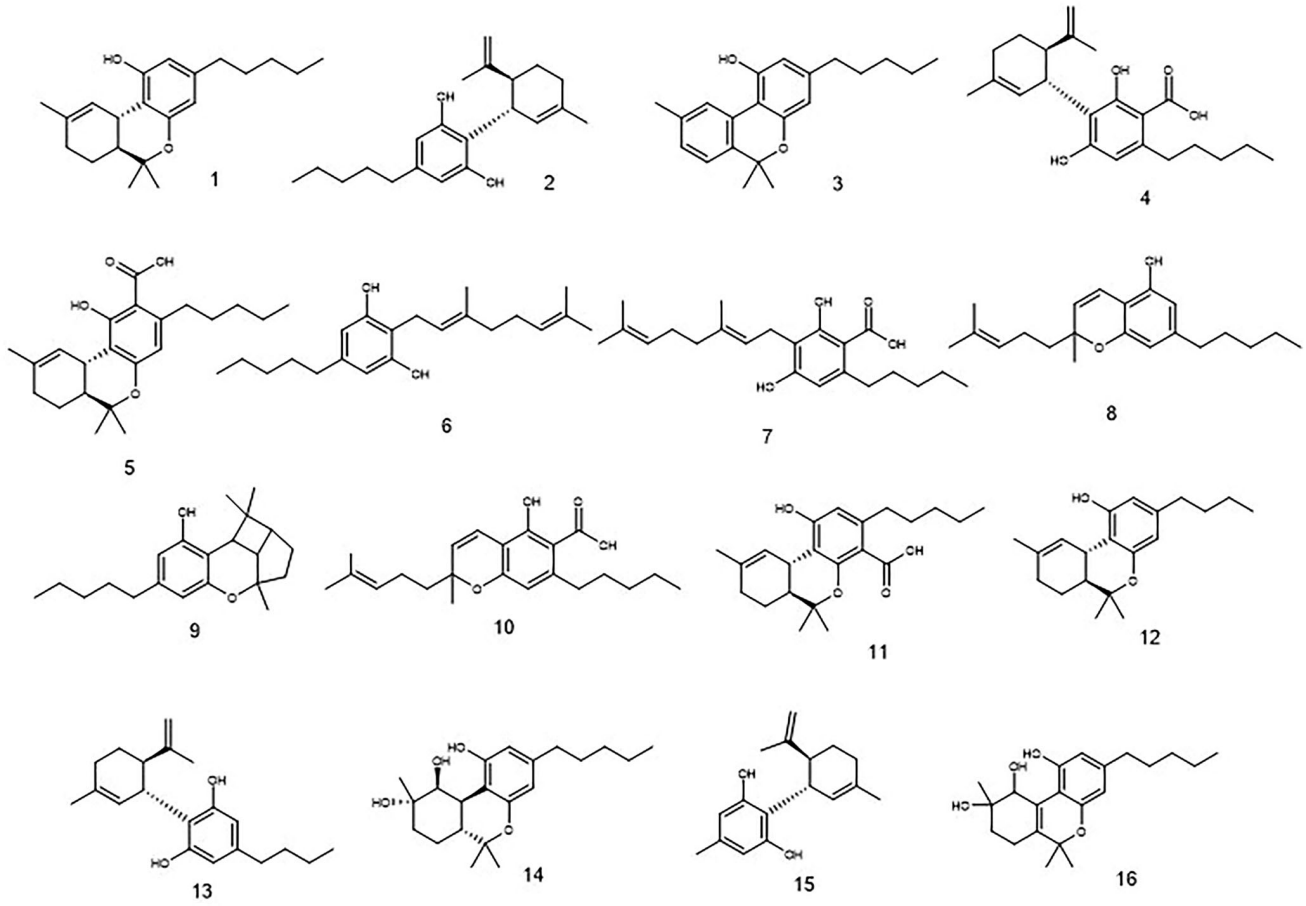
Chemical structures and formulas of compounds detected in *T. micranthum*. **1**, D^9^‐THC, C_21_H_30_O_2_; **2**, CBD, C_21_H_30_O_2;_ **3**, CBN, C_21_H_26_O_2_; **4**, CBDA, C_22_H_30_O_4;_ **5**, THCA‐A, C_22_H_30_O_4_; **6**, CBG, C_21_H_32_O_2_; **7**, CBGA, C_22_H_32_O_4_; **8**, CBC, C_21_H_30_O_2_; **9**, CBL, C_21_H_30_O_2_; **10**, CBCA, C_22_H_30_O_4_; **11**, THCA‐B, C_22_H_30_O_4_; **12**, THC‐C4, C_20_H_28_O_2_; **13**, CBC‐C4, C_20_H_28_O_2_; **14**, Cannabiripsol, C_21_H_32_O_4_; **15**, CBD‐C1, C_17_H_22_O_2_; **16**, CBT, C_21_H_30_O_4_.

**FIGURE 3:**
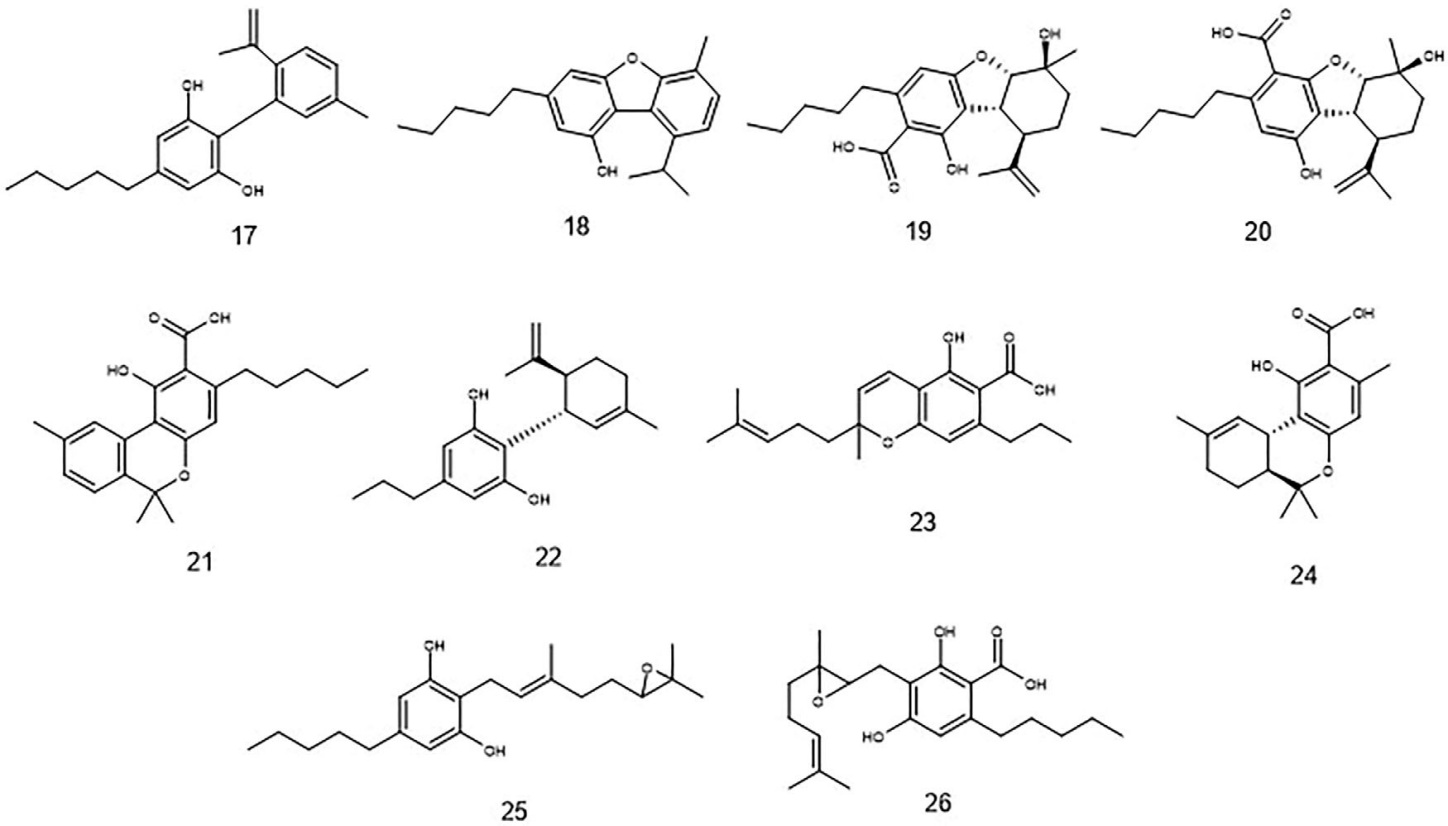
Chemical structures and formulas of compounds detected in *T. micranthum*.**17**, CBND, C_21_H_26_O_2_; **18**, CBF, C_21_H_26_O_2_; **19**, CBEA‐A, C_22_H_30_O_5_; **20**, CBEA‐B, C_22_H_30_O_5_; **21**, Cannabinolic acid, C_22_H_26_O_4_; **22**, CBDV, C_19_H_26_O_2_; **23**, CBCVA, C_20_H_26_O_4_; **24**, THCA‐C1A, C_18_H_22_O_4_; **25**, 6',7'‐epoxy Cannabigerol, C_21_H_32_O_3_; **26**, 6',7'‐epoxy Cannabigerolic acid, C_22_H_32_O_5_.

Twenty‐six cannabinoids were identified, among which CBN, CBG, CBC, and CBCA stand out (Figures 2 and 3). CBN and CBG were identified through targeted analysis. Using the standard fragmentation approach, it was possible to infer the identity of three constitutional isomers of cannabinoids CBC, CBL, and CBCA. By cross‐referencing data from the literature with Full MS analysis, we inferred the identity of an additional 17 cannabinoids. This is the first time these substances have been reported in *T. micranthum*.

Several important cannabinoids present in Cannabis plants, extracts, and medicinal formulations are described as not biosynthesized. They are produced from decarboxylation of the biosynthesized carboxylic acid precursors or by several sequential isomerization and oxidation steps. Some of these processes are natural, and the substances are observed in the plant tissue. Indeed, in the production of Cannabis oily medicinal formulations, for example, a decarboxylation step after extraction, called activation, is performed to increase THC or CBD amounts.

**FIGURE 4:**
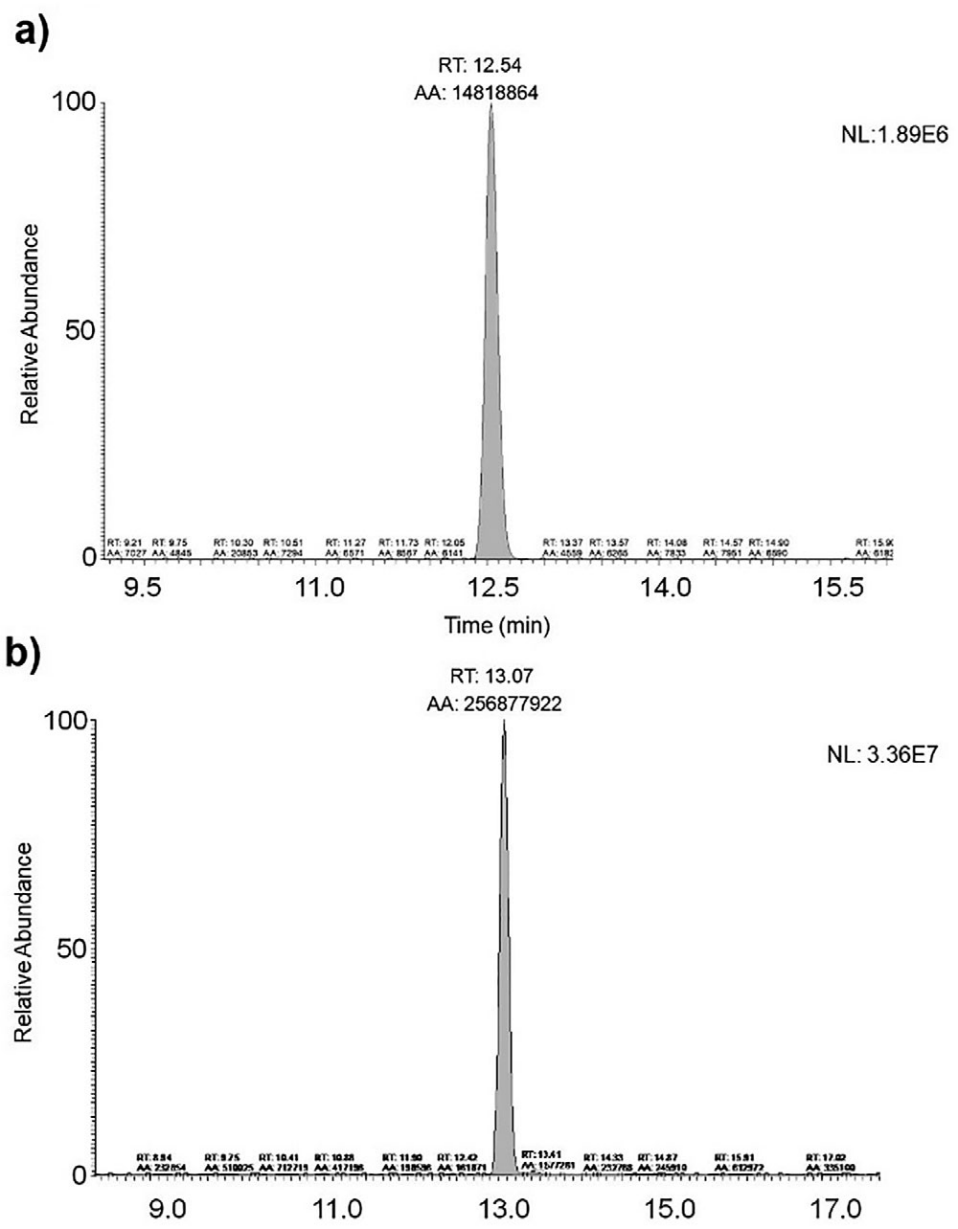
Chromatograms of fragment ion extracted for CBG, CBN, and their retention time. (a) Chromatogram of the reference material of CBG (Rt = 12.54 min); (b) Chromatogram of the reference material of CBN (Rt = 13.07 min).

The targeted approach allowed the identification of CBN and CBG in extracts of *T. micranthum* (Figure 4) by comparing the retention time (RT) and the relative abundance of the diagnostic ions obtained by PRM acquisition of the analyte detected in the sample with those in a reference specimen analyzed in the same analytical sequence. This approach is currently applied for confirmation of analytes for doping control purposes [21].

CBN is a cannabinoid that is described as not naturally being biosynthesized in the plant but produced by the degradation of THC [18]. In ESI‐positive mode, CBN (*m/z* 311) provides a product ion *m/z* 223 (80 Da loss), probably given by a CBN‐dehydrated without a lateral pentyl group and *m/z* 241 (70 Da loss) due to aliphatic 5‐carbon chain cleavage [20]. The *m/z* 195 was monitored consequential of a resorcinol moiety and one carbon atom [22] or sequential pentyl lateral chain and two methyl group losses of a dehydrated CBN [19].

CBG and its precursor, cannabigerolic acid (CBGA), are the first biogenic cannabinoids biosynthesized in the plant [23]. CBGA undergoes decarboxylation to form CBG. In *C. sativa*, this reaction is described as non‐enzymatic and can occur spontaneously during the storage, extraction, or purification of this compound from the plant [24]. In positive mode, CBG (*m/z* 317) provides a product ion *m/z* 193 (124 Da loss), corresponding to the olivetol moiety with the ortho‐methyl group. Subsequently, olivetol undergoes a neutral loss of 70 Da (*m/z* 193–123), corresponding to the side chain of the molecule (C_5_H_10_, pentene), resulting in the detection of the fragment [C_7_H_6_O_2_+H]^+^.

To estimate the concentration of CBN and CBG in the analyzed plant parts, a comparison was made considering the areas of the reference material, which was prepared in methanol/hexane (9:1) at a concentration of 3.16 × 10^−1 ^µg/g, and the plant extracts, as shown in Table 1.

**TABLE 1:** Estimated concentration of cannabinol (CBN) and cannabigerol (CBG) in plant parts.

	CBN (µg/g)	CV%	CBG (µg/g)	CV%
Reference material	3.16E‐01	—	3.16E‐01	—
Green fruits	n.d.	—	1.23E‐02	—
Inflorescences	1.76E‐03	11.34	1.76E‐01	10.32
Leaves	3.57E‐03	9.84	5.49E‐01	10.04
Branches	n.d.	—	n.d.	

n.d. = not detected.

The content of cannabinoids, such as THC, varies in the different parts of the *C. sativa* plant. In general, this content decreases from the uppermost part of the plant, being most abundant in the inflorescences, followed by the leaves. Its presence in the seeds is still a subject of debate [25]. In *T. micranthum*, it is also observed. In Table 1, it is possible to observe that CBG is found in the highest quantity in the leaves, followed by the inflorescences, and in smaller amounts in the fruits.

CBG was not identified as a major constituent of *C. sativa* during the first studies on this plant, but varieties enriched in this compound have recently been generated by hybridization [26]. Based on the concentration values presented in Table 1, CBG was confirmed as the most abundant cannabinoid in *T. micranthum*. This finding highlights the potential of this plant as a producer of a cannabinoid with significant pharmacological activities. CBG is a powerful antagonist of the menthol receptor TRPM8, a target of relevance for prostate cancer, potently activates α‐2 adrenergic receptors, and inhibits with moderate potency 5HT1A serotonin receptors [27]. The activation of α‐2 receptors inhibits the liberation of catecholamine and has been associated with sedation, muscle relaxation, and analgesia [28].

The second approach (Group 2) of CPS tools involves the search for cannabinoids based on the fragmentation pattern and characteristic fragments for specific types of cannabinoids, using PRM as a product ion scan‐like in the quadrupole‐orbitrap mass spectrometer. To achieve this, a classification system was used, based on double bond equivalent (DBE) and average molecular weight (Mw) for the cannabinoids [29], as described in Table 2.

**TABLE 2:** Classification system of the cannabinoids studied in this work.

Molecular formula/double bound equivalent (DBE)	Average molecular weight (Da)	Cannabinoids
C_21_H_26_O_2_/9	310	CBD, CBN, and CBF
C_21_H_30_O_2_/7	314	CBD, CBC, CBL, Δ^9^‐*trans*‐THC, Δ^9^‐*cis*‐THC and Δ^8^‐THC
C_22_H_30_O_4_/8	358	CBDA, CBCA CBLA, Δ^9^‐THCA A or B), and Δ^8^‐THCA

**FIGURE 5:**
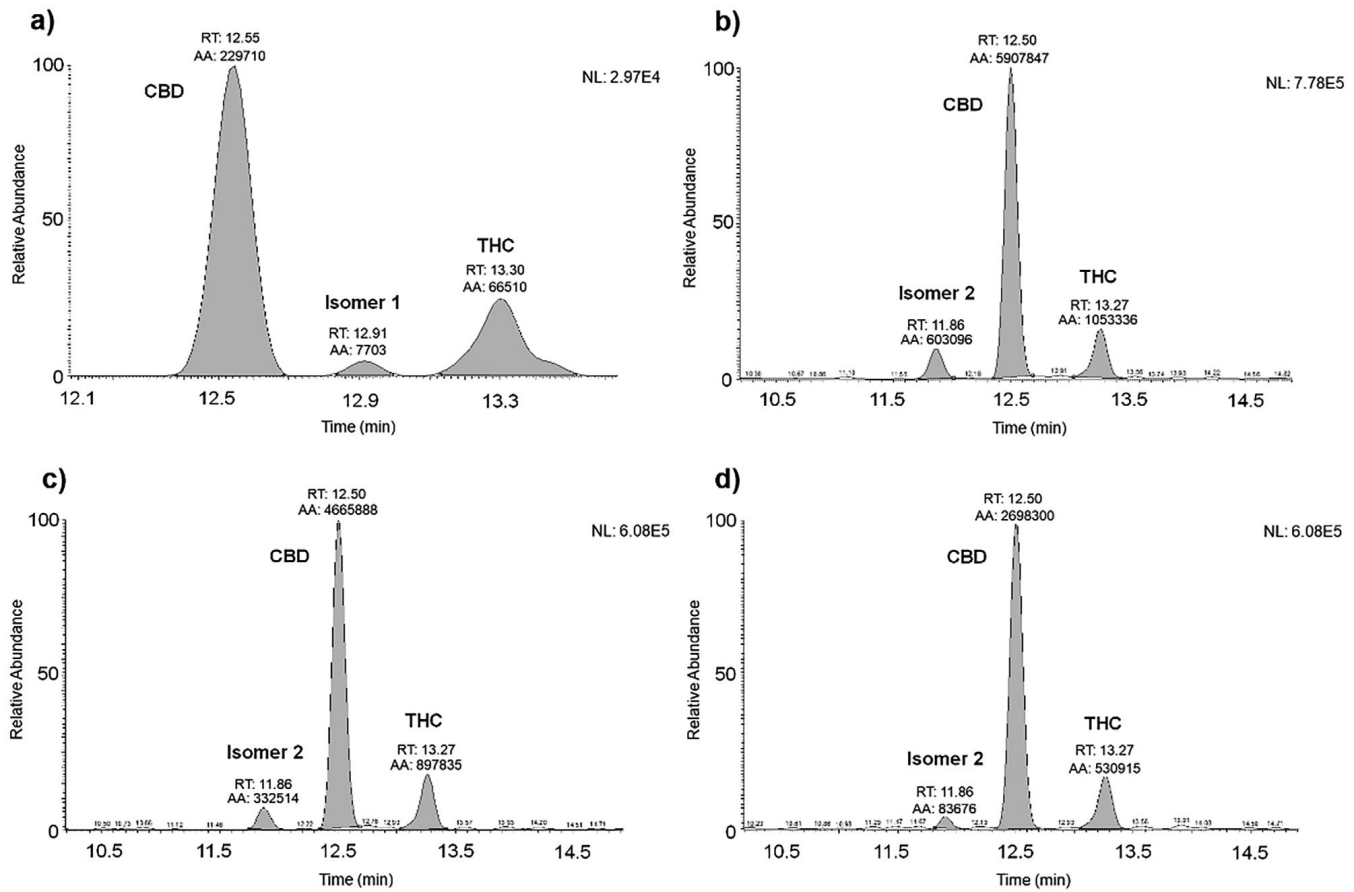
Chromatograms of fragment ions extracted for constitutional isomers and their retention time in *T. micranthum* parts. (a) Isomer 1 (Rt = 12.97 min) in inflorescences extract; Isomer 2 (Rt = 11.86 min) in fruits (b), inflorescences (c), and leaves (d) extracts.

This classification system was used for the guided search for cannabinoid isomers. Cannabinoids with a DBE of 7 and molecular formula C_21_H_30_O_2_, present a pseudomolecular ion *m/z* 315.2318 ([M + H]^+^) and a characteristic fragment *m/z* 193.1223 (base peak) corresponding to olivetol [C_12_H_16_O_2_ + H]^+^. It is possible to observe an isomer called 1, presenting a coefficient of variation of 9.72% (Figure 5a), which elutes between CBD and THC, present only in the inflorescences. Another isomer, referred to as 2 (CV = 10.21%), with a retention time of 11.86 min, elutes before CBD, was observed in all parts of the plant (Figures 5b–d). Table 3 presents the area values found for isomers 1 and 2 in the extracts of *T. micranthum*.

**TABLE 3:** *m/z* evaluated, cannabinoid suggested, molecular formula, and concentration value in ESI mode.

Suggested cannabinoid	Molecular formula [M]	*m/z* [M‐H]^−^	*m/z* [M + H]^+^	Concentration and CV%					
				µg/g	CV%	µg/g	CV%	µg/g	CV%
				Fruits		Inflorescences		Leaves	
Isomer 1	C_21_H_30_O_2_		315.2318			7.03E‐06	11.87		
Isomer 2	C_21_H_30_O_2_		315.2318	1.23E‐06	14.45	1.16E‐05	14.32	1.12E‐05	11.82
CBCA	C_22_H_30_O_4_	357.2071		6.17E‐04	9.91	6.11E‐04	11.15	4.37E‐04	10.33
Canabinodiol or canabifuran	C_21_H_26_O_2_	309.1869	n.a.	1.18E‐06	13.74	1.20E‐05	15.23	1.17E‐05	15.37
Canabielsoic acid A or B	C_22_H_30_O_5_	345.2080	n.a.	n.d.		4.46E‐05	10.46	4.53E‐05	10.17
Cannabinolic acid A	C_22_H_26_O_4_	353.1774	n.a.	4.09E‐06	14.82	3.34E‐06	12.01	1.61E‐05	14.25
Canabigerolic acid	C_22_H_32_O_4_	367.1207	n.a.	1.85E‐06	13.60	1.42E‐06	14.13	n.d.	
Cannabitriol	C_21_H_30_O_4_	389.1970	n.a.	n.d.		6.04E‐04	10.13	8.14E‐04	11.93
THCA‐C1	C_18_H_22_O_4_	301.1445	n.a.	n.d.		2.57E‐04	12.59	5.09E‐04	10.46
Cannabiripsol (CBR)	C_21_H_32_O_4_	n.a.	349.2373	6.08E‐04	12.46	3.47E‐03	14.01	4.45E‐01	10.68
6,7‐Epoxy‐CBGA		n.a.	377.2323	n.d.		4.26E‐03	11.82	5.44E‐02	11.02
6,7‐Epoxy‐CBG	C_21_ H_32_ O_3_	n.a.	333.2424	4.60E‐03	11.27	2.44E‐02	8.56	4.60E‐02	11.43
CBD‐C1	C_17_H_22_O_2_	n.a.	259.1693	2.31E‐02	14.26	2.53E‐02	12.06	1.21E‐02	14.27
CBDV	C_19_H_26_O_2_	n.a.	287.2006	2.43E‐02	12.34	4.50E‐02	8.01	4.78E‐01	10.18
CBD‐C4	C_20_H_28_O_2_	n.a.	301.2162	n.d.		4.49E‐03	11.13	5.55E‐02	10.41
THC‐C4	C_20_H_28_O_2_	n.a.	301.1803	3.55E‐02	13.16	2.63E‐02	11.58	6.23E‐01	10.32
THCA‐C4	C_22_H_30_O_4_	n.a.	345.2070	4.28E‐04	14.11	1.24E‐02	14.92		
CBCVA	C_19_H_26_O_2_	n.a.	331.1904	7.12E‐04	13.92	n.d.		6.33E‐03	13.05

n.a. = not applicable. n.d. = not detected.

The CBG reference material was used to estimate the concentration of isomers 1 and 2, and all other substances identified in this study, presented in Table 3. The amount of isomer 2 in the fruits is higher than in the inflorescences and much lower in the leaves. Similarly, it is possible to observe that the ratio of isomer 2 areas between the fruits and inflorescences is almost 2, meaning that this compound is present in the fruits at almost double the amount found in the Inflorescences. Observing the fruits/inflorescences ratio, there is a significant discrepancy in the presence of this analyte in these parts of the plant, with it being less present in the leaves.

To verify the presence of isomers related to the cannabinoids, C_22_H_30_O_4_ with a DBE of 8, the precursor ion *m/z* 357.2071 was extracted from the Full MS acquisition data (Material S1–S3). A peak was observed with a retention time of 14.25 min, eluting immediately after THCA‐A. To infer the identity of this peak, PRM acquisition was conducted, using the precursor ion and filtering by the fragment *m/z* 313.2177, characteristic of both CBDA and THCA‐A. The fragments *m/z* 311.2022 and *m/z* 339.1970, both exclusively present in the fragmentation pathway of CBDA, were used as mass filters while maintaining the precursor ion (Material S4).

Considering the fragmentation profile observed in the peak with a retention time of 14.25 min, based on the *m/z* 339.1970 fragment present in CBDA's fragmentation pathway, it is possible to exclude any relationship with THCA. The fragmentation pathway of THCA‐A does not involve the formation of *m/z* 339.1970. In this molecule, a neutral loss of 44 Da (CO_2_) occurs from *m/z* 357, forming the deprotonated THC ion [C_21_H_30_O_2_‐H]^−^. The peak with a retention time of 14.26 min and precursor ion *m/z* 357.2071 can lose 44 Da (CO_2_), forming *m/z* 313.2177 [C_21_H_30_O_2_‐H]^−^, or 18 Da (H_2_O), forming *m/z* 339.1970. In the next step, the ion *m/z* 339.1970 undergoes a neutral loss of m/z 28 Da (CO), generating the fragment *m/z* 311.2022 [C_21_H_28_O_2_‐H]^−^. This fragmentation pattern is characteristic of CBDA and CBCA. This evidence, along with the verification of the presence of two constitutional isomers of CBD and THC with a retention time of 11.86 min and 12.97 min, suggests that these are the cannabinoids cannabichromenic acid (Rt = 14.25 min), cannabichromene (Rt = 11.86 min) and cannabicyclol (Rt = 12.97 min). CBC and CBL are formed from the CBCA decarboxylation, and CBC is subsequently converted to CBL by photochemical means [30].

This evidence is corroborated by the concentration presented in Table 3. Since the transformation of CBC into CBL occurs via photochemical processes, it is quite reasonable not to find CBL in the fruit but to detect it in the inflorescences. The area ratio of the isomer referred to as 2 between the inflorescences and the leaf is approximately 4, which explains the absence of its conversion product in this part of the plant.

CBC is a less well‐known cannabinoid compared to THC and CBD, but it has a remarkable therapeutic profile. Plants with a high CBC content result from the selection of recessive genes through multiple crosses between different chemovariants [31]. This compound has significant therapeutic potential, acting as an analgesic and anti‐inflammatory. Additionally, CBC has a moderate affinity for CB2 receptors, which contributes to its anti‐inflammatory properties. CBC can inhibit the reuptake of anandamide, prolonging the effects of this endocannabinoid in the body and contributing to pain relief and mood improvement [32]. Studies indicate that CBC may promote neurogenesis by encouraging the growth of new brain cells, which is particularly relevant in neurodegenerative conditions [33]. This set of properties positions CBC as a promising candidate for future research and clinical applications.

In the last part, data from the literature were used to search for cannabinoids using HRMS, which ensures a superior level of mass accuracy and allows for identifying a greater number of compounds compared to other techniques. Through Full MS mode analysis, many substances could be observed. Table 3 includes the assignments given to these masses in ESI negative and positive mode, the cannabinoid putative, the molecular formula, and the area found in each part of the analyzed plant. The coefficient of variation for all measurements was below 15% for true triplicates performed on different plant samples. Values such as these demonstrate the robustness of the proposed CPS, from extraction to analysis.

The results obtained through the CPS confirm the presence of various cannabinoids in the species *T. micranthum*. It is possible to state that the biosynthesis of cannabinoid compounds is not exclusive to the *Cannabis* genus, being probably related to the Cannabaceae family.

Several clinical reports in the literature highlight the positive health effects of treatments using Cannabis extracts and derivatives. Its use in cancer patients has been reported for the prevention of nausea and pain resulting from chemotherapy, as well as for the restoration of appetite. It is also being studied for cases of anorexia associated with HIV/AIDS [1, 10]. Additionally, antitumor activities have been observed in both in vitro and in vivo models, showing potential tumor cell growth‐retarding and inhibitory effects [10]. Numerous reports also describe its analgesic effects on neuropathic pain, along with documented preclinical studies on its anticonvulsant and anti‐epileptogenic effects [2]. Finally, there is a growing body of literature reporting its effectiveness in palliative care and symptom management for clinical conditions such as fibromyalgia, epilepsy, sleep disorders, multiple sclerosis, gastrointestinal reflux, irritable bowel syndrome, hypertension, schizophrenia, generalized anxiety disorder, and others [1, 2]. The present study identified the presence of 26 cannabinoids in *T. micranthum*, which opens up possibilities for investigating its use from a wide pharmacological perspective.

## 3 Conclusions

In the present work, an analytical method was developed that allowed the detection of several cannabinoids, called CPS. Applying this method to *T. micranthum* extracts, the identification of 26 cannabinoids was achieved. Of these, two cannabinoids were identified by reference material, and 18 were putatively annotated, according to mass experiments and exact mass. It is noteworthy that for the first time several cannabinoids, which to the best of our knowledge have never been reported, have been identified in *T. micranthum*.

## 4 Experimental

### 4.1 General

A Dionex Ultimate 3000 ultra‐high performance liquid chromatography (UHPLC) system coupled to a QExactive Plus hybrid quadrupole Orbitrap mass spectrometer (Thermo Fisher Scientific, Bremen, Germany) equipped with an ESI source was used. Separation was performed in a reversed‐phase column (kinetex 2.6 µm PS C18, 100Å, 100 mm x 2,1 mm; 2,6 µm), at 40°C, constant flow rate of 300 µL/min and injection volume of 5 µL. A gradient chromatographic run started at 5% of mobile phase B (methanol with 0.1% formic acid) and 95% of mobile phase A (water with 5 mM ammonium formiate and 0.1% formic acid). Mobile phase B was increased to 10% at 1.0 min, then to 25% at 2 min, and then 90% at 10 min. After reaching 100% of B at 14 min and maintaining this ratio until 16 min, the initial chromatographic condition was restored from 16.1 to 20.0 min.

The LC effluent was pumped to the mass spectrometer operating in a positive and negative ESI mode, calibrated daily with a manufacturer's calibration solution (Thermo Fisher Scientific, Bremen, Germany). ESI parameters were further optimized, with the final setup: spray voltage of 2.9 kV, S‐lens voltage of 80 V, the capillary temperature of 380°C, auxiliary gas heater temperature of 350°C, nitrogen sheath, auxiliary, and sweep gas were set at 30, 10, and 1 arbitrary unit, respectively. Full‐scan data were acquired in a range of *m/z* 70–1050 at a resolution of 70 000 full widths at half maximum (FWHM), automatic gain control (AGC) of 1 × 10^6^, and maximum injection time (IT) of 100 ms.

Targeted MS‐based approaches were performed using the PRM, the precursor ions were fixed at a resolution of 17 500 FWHM, AGC of 1 × 10^6^, maximum IT of 100 ms, and quadrupole isolation window of *m/z* 2.

Data was acquired and processed using Thermo Scientific TM Trace Finder TM 4.1 software (Thermo Fisher Scientific, Austin, TX, USA), with a ± 6 ppm mass tolerance. Exact mass of *m/z* 311.23186 ([M + H]^+^) to CBN, and *m/z* 317.24750 ([M + H]^+^) to CBG. The LC effluent was pumped to the mass spectrometer operating in a positive and negative ESI mode, calibrated daily with a manufacturer's calibration solution (Thermo Fisher Scientific, Bremen, Germany).

To detect and quantify CBN and CBG, in the plant parts, the standard mixtures were prepared in methanol at a concentration of 3.16 × 10^−1^ µg/g.

The detection limit established by the PRM technique at MS was 5.2 × 10‐4 µg/g for CBD and 3.7 × 10‐4 µg/g for THC, 1.4 × 10‐3 µg/g for CBDA and 1.0 × 10‐3 µg/g for THCA A per gram of extract. The estimated limit of quantification established by PRM was 1.7 × 10^−3^ µg/g for CBD and 1.2 × 10^−3^ µg/g for THC, 4.7 × 10^−3^ µg/g for CBDA and 3.5 × 10^−3^ µg/g for THCA A per gram of extract. In the PRM approach, precursor ions of *m/z* 315.23184 [M + H]^+^ (CBD and THC), and *m/z* 357.20713 [M‐H]^−^ (CBDA and THCA A) were fragmented in a higher energy collisional dissociation (HCD) cell with (N)CE of 40%.

### 4.2 Plant Material

The plant material was collected in Parque Nacional da Tijuca, close to the Pedra da Gávea, Rio de Janeiro‐RJ, Brazil, on July 10th, 2024, identified by one of the authors, the biologist Ricardo Finotti, with a voucher deposited at RB 648300 Herbarium and also registered at SISGEN under the number AE058C8.

### 4.3 Extraction

Ten grams of plant parts, including branches, leaves, and inflorescences, were extracted using a methanol/hexane solvent mixture in a 9:1 (v/v) ratio. One gram of macerated green fruit was weighed, and the same solvent mixture was used for extraction. The plant materials immersed in the solvents were subjected to extraction under agitation in a shaker at 150 rpm for 12 h. The resulting organic extract was centrifuged at 4000 rpm for 20 min and evaporated under nitrogen flow at 40°C. The dry residue was reconstituted with methanol to obtain a final concentration of 10 µg/mL. The extracts were vortexed for 30 s, 1.5 mL was transferred to an Eppendorf tube, and centrifuged at 13 000 rpm for 15 min at 15°C. The supernatant was transferred to a vial and injected into LC‐HRMS/MS. This procedure was prepared in triplicate, using a new sample of the plant. Each replicate was injected only once. The media, standard deviation, and coefficient of variation were calculated for each analyte.

## Author Contributions

All authors analyzed the data and contributed equally to the study.

## Conflicts of Interest

The authors declare no conflicts of interest.

## Supporting information



Supporting information for this article is available on the WWW under https://doi.org/10.1002/MS‐number.

## Data Availability

The authors have nothing to report.
