# Supplementary material for: Cannabinoids From Trema micranthum (L.) Blume (Cannabaceae): A Cannabinoid Profiling System
Source: Chem Biodivers. 2025 May 22;22(10):e00208. doi: 10.1002/cbdv.202500208 (PMC12533798; doi:10.1002/cbdv.202500208)
Supplement: Supplementary file 1 — Supporting information for this article is available on the WWW under https://doi.org/10.1002/MS‐number. [file CBDV-22-e00208-s001.docx]

***Supplementary Material***

**Figure S1.** HRMS fragmentation spectrum of CBDA.

**Figure S2.** HRMS fragmentation spectrum of THCA A.

**Figure S3.** HRMS fragmentation spectrum of CBCA.

**Figure S4**. Fragmentation pattern approach of group 3cannabinoids isomer, C_22_H_30_O_4_ with a DBE of 8, the precursor ion *m/z* 357.2071


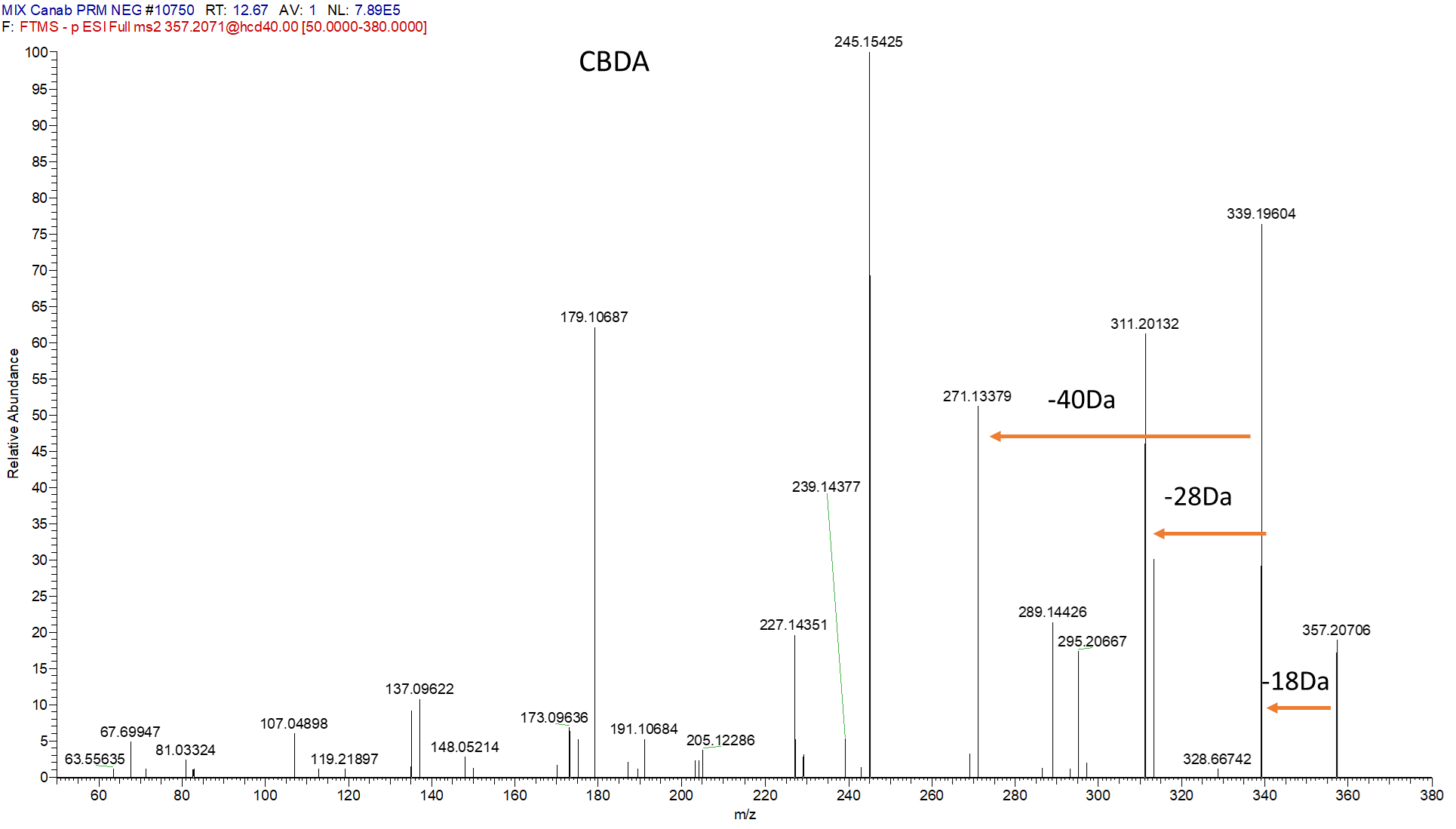


**Figure S1.** HRMS fragmentation spectrum of CBDA.


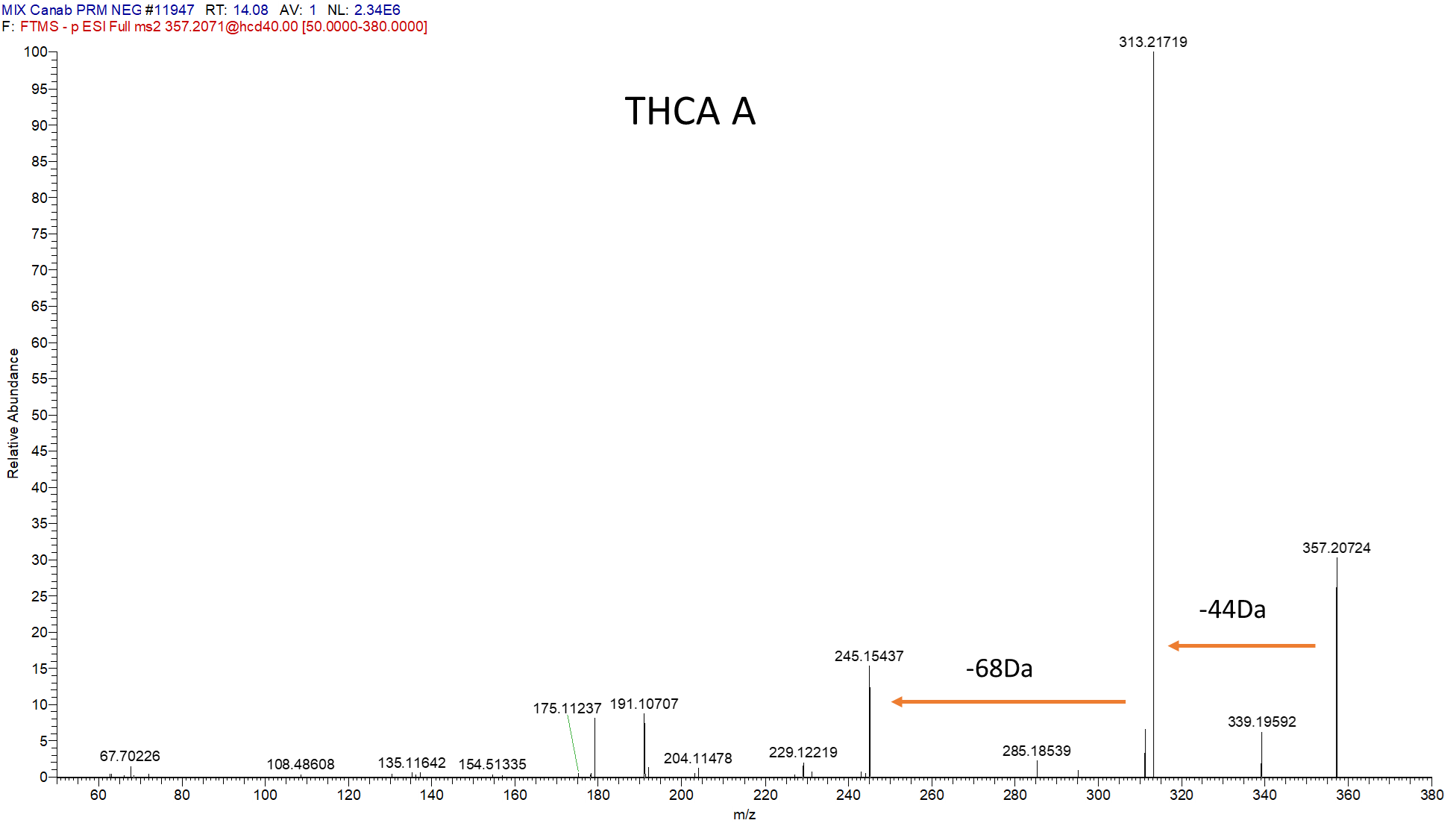


**Figure S2.** HRMS fragmentation spectrum of THCA A.

-44Da

-18Da

**Figure S3.** HRMS fragmentation spectrum of CBCA.


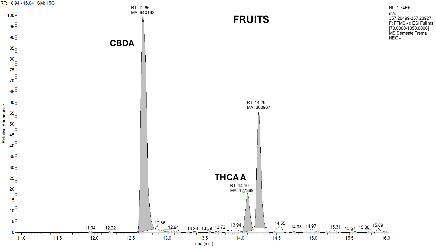

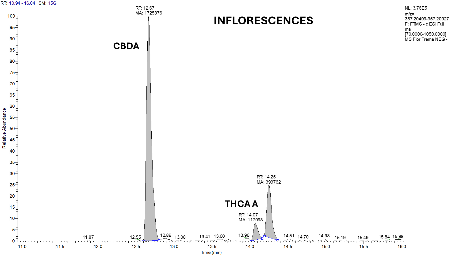

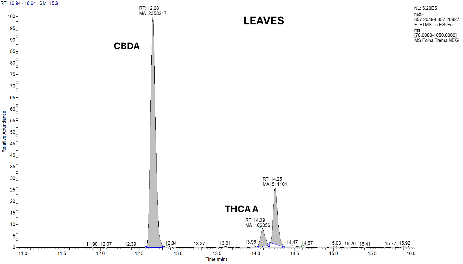


**C**

**B**

**A**


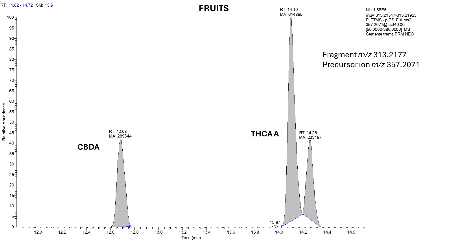

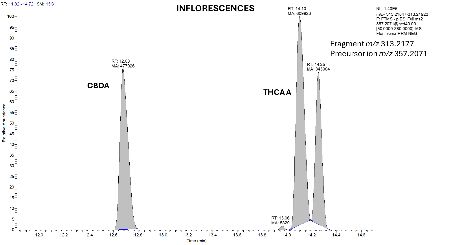

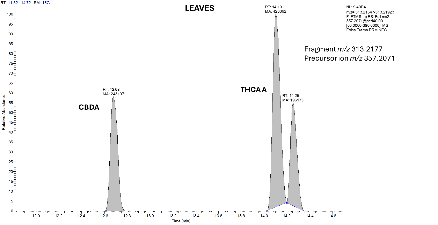


**G**

**H**

**I**

**F**

**E**

**D**


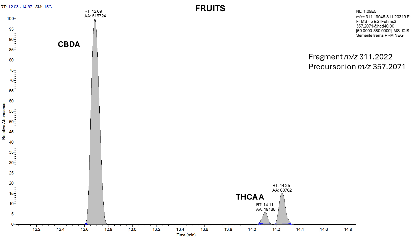

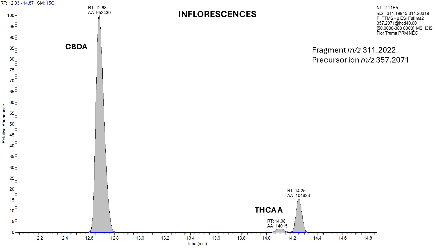

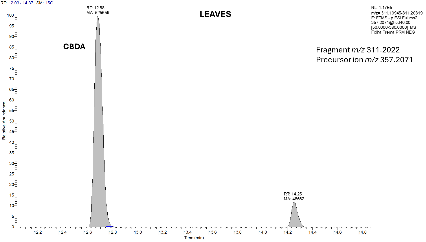


**L**

**K**

**J**


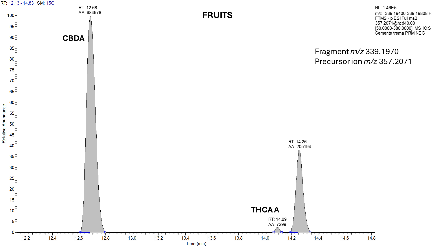

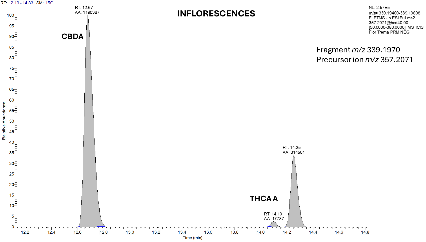

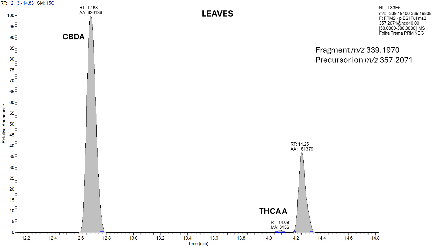


**Figure S4**. MS data from *Trema* *micranthum* parts. **A**, **B** and **C**: Extracted-ion chromatograms for *m/z* 357.2071. **D**, **E** and **F:** Extracted-ion chromatograms of fragment ion extracted *m/z* 313.2177. **G**, **H** and **I:** Chromatograms of fragment ion extracted *m/z* 311.2022. **J**, **K** and **L:** Extracted-ion chromatograms from *m/z* 339.1970.
